# Efficacy of robot-assisted stereotactic aspiration in moderate basal ganglia hemorrhage: a retrospective cohort study

**DOI:** 10.3389/fsurg.2026.1777756

**Published:** 2026-06-17

**Authors:** Guangjie Liu, Jiachuan Liu, Shen Xu, Manmiao Hu, Linsen Li, Chunlin Wang

**Affiliations:** Department of Neurosurgery, The 901st Hospital of the Joint Logistics Support Force of PLA, Hefei, China

**Keywords:** drainage, fragmentation and suction, minimally invasive, puncture, robot-assisted, spontaneous intracerebral hemorrhage, stereotactic

## Abstract

**Objective:**

To study the clinical efficacy of robot-assisted stereotactic hematoma puncture and fragmentation in the treatment of moderate basal ganglia hemorrhage.

**Methods:**

A retrospective analysis was conducted on 64 cases of basal ganglia hemorrhage (BGH) patients admitted to the 901st Hospital of the Joint Logistics Support Force from October 1st 2023 to June 1st 2025. The experimental group (*n* = 34) received robot-assisted stereotactic puncture and aspiration drainage, while the control group (*n* = 30) underwent traditional manual CT-guided hematoma puncture and catheter placement for aspiration drainage. The two groups were compared in terms of operative time, hematoma clearance rate, number of urokinase instillations, drainage tube retention duration, as well as NICU hospitalization duration, mannitol usage duration, 72-hour cerebral edema volume, Glasgow Coma Scale (GCS) score, National Institutes of Health Stroke Scales (NIHSS) score, Activities of Daily Living (ADL) score at 1 month postoperatively, modified Rankin Scale (mRS) score at 3 months postoperatively, and complication rates.

**Results:**

Compared with the control group, the experimental group demonstrated statistically significant improvements during treatment, including reduced pulmonary infection rates, shorter NICU hospitalization duration, decreased mannitol usage duration, reduced 72-hour cerebral edema volume, significantly improved postoperative GCS and NIHSS scores, better ADL scores at 1 month postoperatively, and better (lower) mRS scores at 3 months postoperatively (*P* < 0.05).

**Conclusion:**

Robot-assisted stereotactic minimally invasive puncture and aspiration for basal ganglia hemorrhage catheterization provides more precise and thorough hematoma evacuation, reduces postoperative complication rates, and significantly promotes postoperative neurological recovery.

## Introduction

Spontaneous intracerebral hemorrhage (sICH) is the most common acute and severe type of hemorrhagic stroke, with basal ganglia hemorrhage accounting for the highest proportion. It is a significant cause of death or disability worldwide, with an early mortality rate of 30%–40% (1, 2). The volume of hemorrhage directly determines the severity of the patient's condition. For basal ganglia hemorrhage patients with a volume of 20–40 mL, there is no unified treatment standard domestically or internationally (3, 4). The treatment strategies for this group (conservative, minimally invasive, and craniotomy) are the most controversial. Minimally invasive hematoma puncture and drainage (MIPD) has been widely recognized due to its advantages of high surgical safety, simple operation, and minimal trauma (5–7). However, the accuracy of conventional CT-guided manual puncture, which relies on surface landmarks, is often suboptimal, thereby potentially limiting surgical outcomes. With the advancement of stereotactic navigation, robot-assisted stereotactic techniques have emerged as a promising alternative, offering the potential for greater precision. Although evidence supports the therapeutic value of stereotactic aspiration for ICH (8, 9), the specific advantages of robot-assisted techniques over traditional methods in patients with moderate-volume hemorrhages require further investigation.

In this retrospective study, we compared the outcomes of robot-assisted stereotactic techniques vs. conventional CT-guided manual puncture in patients with sICH (hematoma volume 20–40 mL). We hypothesized that the robotic approach would result in superior hematoma evacuation, better short-term neurological recovery, and fewer complications. Our findings may provide evidence to support the efficacy of robot-assisted stereotactic techniques for this specific patient population.

## Methods

### Design and participants

A retrospective study was carried out to compare the effects of patients received robot-assisted stereotactic and traditional CT-positioned manual minimally invasive puncture aspiration and drainage treating of basal ganglia intracerebral hemorrhage. Patients were assigned to either the robot-assisted group or the control group according to the group they preferred. This study followed the revised Helsinki Declaration in 2013, and all patients and their families signed the informed consent form. Spontaneous cerebral hemorrhage were enrolled from October 1st, 2023, to June 1st, 2025. The inclusion criteria were as follows: (1) Diagnosis of spontaneous intracerebral hemorrhage (ICH) was confirmed according to the 2020 “Guidelines for Diagnosis and Treatment of Spontaneous Intracerebral Hemorrhage” criteria (10); (2) This admission was the first episode of bleeding, with hematoma volume located in the basal ganglia region; (3) Glasgow Coma Scale (GCS) score ≥ 8 and hematoma volume 20–40 mL within 48 h from onset to admission; (4) No coagulation dysfunction; (5) No severe underlying cardiovascular or pulmonary diseases. Exclusion criteria: (1) Patients with multiple episodes of ICH; (2) History of ipsilateral stroke or residual neurological deficits; (3) Coagulation dysfunction, recent use of antiplatelet or anticoagulant medications, or platelet count < 100 × 10⁹/L; (4) Bleeding caused by other etiologies (e.g., arteriovenous malformations, intracranial aneurysms). This study was approved by the Medical Ethics Committee of the 901st Hospital of the Joint Logistics Support Force and complied with the “Helsinki Declaration” (11). All patient families provided informed consent. All surgical procedures in both groups were performed by the authors, who are all qualified and experienced neurosurgeons from the Department of Neurosurgery.

### Interventions

All patients received comprehensive medical management based on the Chinese Multidisciplinary Diagnosis and Treatment Guidelines for Spontaneous Cerebral Hemorrhage (3). Upon admission, both groups underwent thorough preoperative examinations, and no significant contraindications for surgery were identified. Cranial CT angiography (CTA) was routinely performed to rule out intracranial hemorrhage secondary to aneurysms or vascular malformations. All surgical procedures in both groups were performed under general anesthesia with endotracheal intubation.

#### Control group (conventional CT-guided puncture)

The preoperative cranial CT scan was used to plan the puncture target, which was set at the center of the largest cross-section of the hematoma, while cautiously avoiding eloquent cortical areas. Following general anesthesia and standard sterile draping, the puncture site, trajectory, and depth were determined based on external anatomical landmarks and the pre-operative CT measurements. A small scalp incision was made, a burr hole was drilled, and the dura mater was electrocoagulated and incised in a cruciate fashion, followed by cortical electrocoagulation. A Medtronic 3.1 mm drainage tube was then inserted into the hematoma cavity along the pre-determined trajectory. Successful placement was confirmed by the aspiration of bloody fluid. Gentle manual aspiration was performed using a syringe, and aspiration was halted immediately if significant resistance was encountered to avoid rebleeding. The tube was then connected for continuous low-level closed drainage. Postoperative management included strict blood pressure control, sedation and analgesia, and administration of dehydrating agents as needed. For clot lysis, a urokinase regimen was administered through the drainage tube starting 24 h post-surgery, following a confirmatory CT scan showing no active bleeding. The regimen consisted of urokinase (typically 20,000–50,000IU) diluted in 2–3 mL of normal saline, instilled into the hematoma cavity. The drainage tube was then clamped for 1–2 h before being reopened for drainage. This procedure was repeated once or twice daily, as determined by the volume of residual hematoma on follow-up imaging, and continued for up to 3–5 days or until the drainage tube was removed.

#### Observation group (robot-assisted stereotactic puncture)

Preoperatively, patients underwent scalp shaving and the placement of 4–6 adhesive fiducial markers (MARK points). A thin-slice cranial CT scan (slice thickness < 1 mm) was then performed, ensuring full coverage of all markers. The image data was imported into the Remebot neurosurgical robotic system [Remebot, Huake Precision (Beijing) Medical Technology Co., Ltd. China], a frameless stereotactic robot. The robotic system's software was used to: 1) register the fiducial markers; 2) define the target, similarly set at the hematoma's center; 3) plan the optimal trajectory to avoid critical vessels, sulci, and functional areas; and 4) measure the precise depth to the target.

After the induction of general anesthesia, the patient's head was immobilized using a three-pin Mayfield head holder, which was then registered and connected to the robotic system. The robotic arm was moved to its initial position to complete the spatial registration. A secondary registration of the fiducial markers was performed to verify system accuracy; any marker with a registration error > 1 mm was excluded to ensure the final accuracy was ≤ 1 mm. The robotic arm was then automatically guided to the pre-planned entry point, indicating the exact scalp incision site, drilling position, and trajectory. After reconfirming the path and coordinates, a burr hole was drilled. Guided precisely by the robotic arm along the pre-set trajectory, a specialized hemostatic puncture needle was inserted into the hematoma. The hematoma was aspirated by repeatedly rotating the needle and applying gentle suction. Once satisfactory aspiration was achieved, as indicated by reduced resistance and/or aspiration volume consistent with preoperative planning, the hemostatic needle was removed. A drainage tube was then placed into the hematoma cavity through the same trajectory. The catheterization procedure, postoperative management, and the urokinase regimen (initiation time, dosage, frequency, and duration) were identical to those described for the control group.

#### Postoperative assessment

A non-contrast cranial CT scan was routinely performed within 24 h post-surgery for all patients to assess the hematoma clearance rate, verify the position of the drainage tube, and check for any new or worsened intracranial hemorrhage. The volume of the hematoma was calculated with 3D Slicer software. The hematoma evacuation volume (Ve) was defined asthe hematoma volume (Vp) before surgery minus the residual hematoma volume (Vr) at the first brain CT scan after surgery. The hematoma clearance rate (HR) was calculated as (Vp-Vr) = Vp*100%. Subsequent CT scans were performed as clinically indicated to monitor for rebleeding, resolution of edema, and to guide the continuation or cessation of the urokinase regimen.

### Outcomes

The duration of the operation, hematoma clearance rate, frequency of urokinase use, indwelling time of the drainage tube, as well as the length of stay in the NICU during the treatment period, the duration of mannitol use, and the volume of cerebral edema within 72 h as the primary outcomes, In addition, as secondary outcomes, the occurrence of complications were also compared in the two groups. Glasgow Coma Scale (GCS) scores, National Institutes of Health Stroke Scales (NIHSS) scores, Activities of Daily Living (ADL) score one month after the operation and modified Rankin scale (mRS) scores three months after the operation were used to evaluate the recovery of neurological function in patients after the operation.

## Statistical analysis

All the data were analyzed using the statistical software SPSS (version 26, IMB Corporation). The Kolmogorov‒Smirnov test was applied to determine whether the quantitative data met normality. Quantitative data following normal distribution were described as mean ± standard deviation ($\bar{x}$±SD) and compared using an independent sample t-test. Quantitative data not following normal distribution were described as the median (P25, P75) and were compared using the Wilcoxon rank sum test. Qualitative data were described as numbers (percentages) and were compared using the Chi-Square test or the Fisher's exact test. The difference was considered to be statistically significant (*P* < 0.05).

## Results

### Patient clinical characteristics

A total of 181 patients were diagnosed with basal ganglia hemorrhage from October 1st, 2023 to June 1st, 2025. Among them, 72 patients received robot-assisted stereotactic or CT-positioned Catheterization operation treatment.8 patients were excluded due to missing follow-up. Hence, 64 patients were recruited in this retrospective study based on the inclusion and exclusion criteria. A total of 34 patients, comprising 19 males and 15 females, with an average age of 56.09 ± 11.18 years, were in the Robot-assisted group; and 30 patients, comprising 16 males and 14 females, with an average age of 56.13 ± 11.68 years, were in the CT-positioned group. The baseline characteristics between the two groups were similar.

### Surgical parameters in the two groups

#### Comparison of surgical outcomes between the two groups

**Figure 1:**
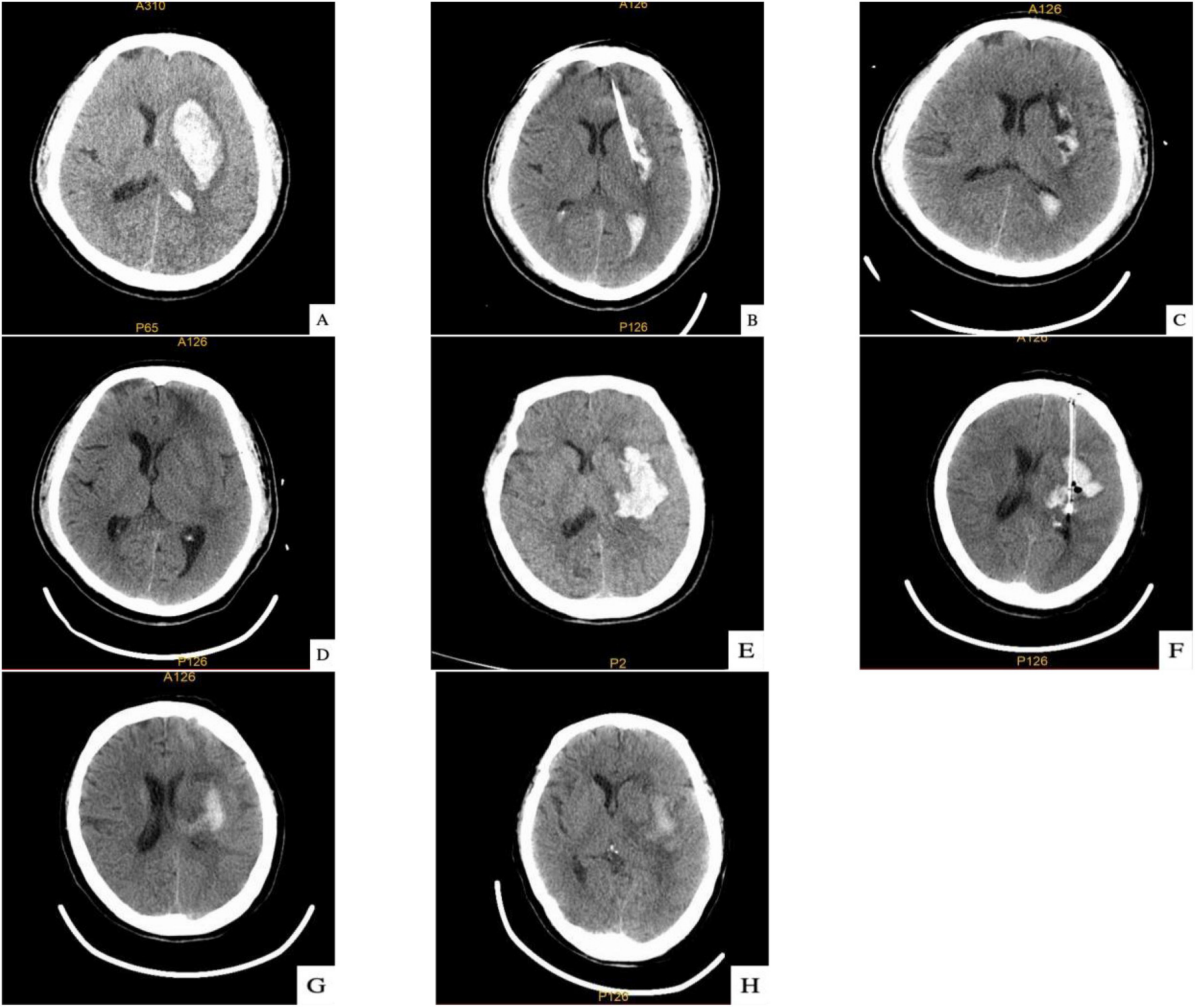
Comparison of pre-and postoperative hematoma volumes and clearance rate between the two groups. Experimental Group **(A)** Preoperative hematoma volume was 29.65 mL; **(B)** Postoperative day 1, hematoma volume was 2.32 mL; **(C)** Postoperative day 2, hematoma volume was 2.18 mL; **(D)** Postoperative day 7, hematoma volume was 1.75 mL, with a hematoma clearance rate of 92.17%. Control Group **(E)** Preoperative hematoma volume was 27.61 mL; **(F)** Postoperative day 1, hematoma volume was 8.51 mL; **(G)** Postoperative day 2, hematoma volume was 7.61 mL; **(H)** Postoperative day 7, hematoma volume was 6.53 mL, with a hematoma clearance rate of 69.18%.

In the control group, CT scanning combined with surface markers was used for manual localization of puncture targets, resulting in lower localization accuracy, greater postoperative residual hematoma volume, and prolonged duration of cerebral edema (Figures 1A–D). In the experimental group, data were obtained after thin-layer cranial CT scanning, and a robotic system was utilized to precisely locate the puncture target. The drainage tube was accurately placed along the long axis of the hematoma cavity, successfully fragmenting and aspirating most of the hematoma. Postoperative residual volume was minimal, and the hematoma was rapidly absorbed, with a significantly shortened duration of cerebral edema (Figures 1E–H). The hematoma clearance rate in the experimental group was significantly improved, the number of urokinase administrations was markedly reduced, and the duration of drainage tube placement in the hematoma cavity was significantly shortened, all of which showed statistically significant differences (*P* < 0.05) (Table 1).

**Table 1:** Comparison of perioperative and treatment-related parameters and complications between the two groups [x ± s/n (%)/M (Q1, Q3)].

	Observation group (*n* = 34)	Control group (*n* = 30)	t/Fisher's value	*P* value
Operative duration (min)	45.2 ± 8.3	41.8 ± 10.1	4.01	0.077
Intraoperative blood loss (mL)	11.5 ± 6.2	12.8 ± 7.5	−0.759	0.451
Hematoma clearance rate, n(%)	91.5 ± 5.1	75.3 ± 6.8	10.67	<0.001
Urokinase dosage frequency	2.1 ± 0.81	2.9 ± 1.20	3.15*	<0.05*
Duration of drainage tube placement (d)	2.2 ± 0.90	3.5 ± 1.31	−4.70	<0.05
Volume of cerebral edema (mL)	8.3 ± 3.2	12.6 ± 4.5	4.50	<0.001
NICU stay duration (d)	5.2 ± 1.8	7.8 ± 2.5	4.62*	<0.001
Mannitol administration duration (d)	6.5 ± 2.1	8.9 ± 2.8	3.85*	<0.001

* Fisher's exact test.

### Treatment related parameters in the two groups

Comparison of relevant parameters between the two groups during treatment. No statistically significant difference was observed in the rebleeding rate between the two groups (*P* > 0.05). Compared with the control group, the experimental group showed a reduced incidence of pulmonary infection, shorter NICU hospitalization duration, decreased mannitol usage time, and a smaller 72-hour cerebral edema volume, with statistically significant differences (*P* < 0.05) (Table 1).

### Complication rates

The observation group reported significantly fewer overall incidence of postoperative complications than in the control group (*P* < 0.05; Table 2). One case of intracranial infection (2.94%) occurred in the observation group, which was confirmed by CSF analysis and resolved completely after intravenous administration of vancomycin and meropenem. One case of rebleeding (2.94%), the bleeding primarily occurred at the puncture site and was absorbed after conservative medical treatment. In the control group, Four case of intracranial infection (13.3%), Three patients experienced postoperative rebleeding (10.0%), Two requiring repeat craniotomy.

**Table 2:** Comparison of complication rates between the two groups [n (%)].

	Control group	Observation group	*χ*^2^ value	*P* value
Stress ulcers	3	2	–	–
Deep vein thrombosis	3	2	–	–
Rebleeding (n)	3	1	–	–
Intracranial infeion (n)	4	2	–	–
pulmonary infection (n)	4	2	–	–
Total incidence	17 (56.6)	9 (26.5)	6.02	0.0014

### Prognosis of patients in the two groups

No statistically significant difference was observed in the NIHSS scores between the two groups at admission (*P* > 0.05). The experimental group showed significantly higher improvements in GCS and NIHSS scores compared to the control group. Specially, On the 7th postoperative day, the improvement in GCS and NIHSS scores in the experimental group was significantly higher than that in the control group, with statistically significant differences (*P* < 0.05) (Table 3). Additionally, the ADL scores and self-care ability ratio at 1 month postoperatively, as well as the modified Rankin scale (mRS) scores at 3 months postoperatively, were all superior in the experimental group compared to the control group, with statistically significant differences (*P* < 0.05) (Table 4).

**Table 3:** Comparison of proportions of GCS improvement ≥ 2 points and degree of NIHSS improvement at 7 days post-operation between the two groups.

Group	Proportion with GCS improvement ≥ 2 points	Degree of NIHSS improvement			
		Marked improvement	Improvement	No change	Deterioration
Observation group (*n* = 34)	70.6% (24/34)	15	12	5	2
Control group (*n* = 30)	46.7% (14/30)	8	10	9	3
*χ*^2^ value	2.12	2.08	0.75	1.32	0.45
*P* value	0.038	0.043	0.457	0.192	0.656

The proportion of patients with a GCS improvement of ≥ 2 points and the degree of NIHSS score improvement were analyzed at 7 days postoperatively. The degree of NIHSS score improvement was defined as follows: a reduction of ≥ 4 points after treatment was considered significant improvement; a reduction of 1–3 points was considered improvement; no change in score was considered stable; and an increase in score was considered deterioration.

**Table 4:** Comparison of Barthel Index and mRS scores between the two groups (X ± S).

Group	*n*	1 months postoperatively		3 months postoperatively	
		Barthel index score	Barthel index score ≥ 60 score (n)	mRS score	mRS score indicates a favorable prognosis (mRS ≤ 3 score, n)
Observation group	34	65.2 ± 12.3	28 (82.4%)	2.1 ± 1.2	29 (85.3%)
Control group	30	58.5 ± 14.6	19 (63.3%)	2.9 ± 1.5	20 (66.7%)
t/*x*^2^ value		2.05	2.12	2.38	2.08
*P* value		0.045	0.038	0.021	0.042

## Conclusions

Robot-assisted stereotactic minimally invasive puncture and aspiration for basal ganglia hemorrhage catheterization provides more precise and thorough hematoma evacuation, reduces postoperative complication rates, and significantly promotes postoperative neurological recovery.

## Discussion

This study revealed through retrospective analysis that robot-assisted stereotactic aspiration for moderate basal ganglia hemorrhage significantly improved hematoma clearance, reduced perioperative morbidity, and enhanced short-term neurological and functional recovery compared to conventional CT-guided puncture.

Our findings align with the growing body of evidence supporting minimally invasive surgery for ICH, as highlighted by the MISTIE III trial, which demonstrated that minimally invasive surgery plus thrombolysis can reduce mortality and improve functional outcomes when a low residual hematoma volume is achieved (12). Our study extends these findings by showing that robot-assisted aspiration can achieve a high degree of hematoma clearance (> 90% in our series) safely, potentially maximizing the benefits of the minimally invasive approach.

The superior neurological outcomes (GCS, NIHSS, ADL, mRS) in the robot-assisted group are likely multifactorial. The primary driver is the significantly higher hematoma clearance rate, which promptly alleviates mass effect and reduces the neurotoxic burden from blood breakdown products (13, 14). This is supported by the observed reduction in 72-hour cerebral edema volume and mannitol usage. Stereotactic robots can precisely locate and perform hematoma aspiration, a technique widely validated in clinical practice (15, 16). By flexibly designing the puncture pathway along the hematoma's long axis, the procedure enables accurate lesion localization while avoiding damage to critical intracranial structures such as cerebral sulci, ventricles, and visible blood vessels (17, 18). During the procedure, most of the hematoma can be safely and effectively fragmented and aspirated, achieving a clearance effect comparable to traditional microscopic or endoscopic surgery but with less damage (12).

The high precision and safety profile of robot-assisted systems in our study are consistent with recent reports on robotic stereotactic aspiration for deep-seated ICH (8, 19). The study also revealed that due to the higher hematoma clearance capability in the robot-assisted group, the frequency of urokinase administration and the duration of drainage tube placement were significantly shorter (20). Additionally, the robot-assisted group exhibited lower incidence of postoperative complications such as intracranial infections and rebleeding, suggesting that robotic stereotactic hematoma drainage surgery is safe, which aligns with findings from other scholars (21, 22).

This improvement markedly reduced secondary damage to surrounding brain tissue caused by degradation products from extensive hematoma liquefaction (23, 24), resulting in a substantial reduction in brain edema volume during the 72-hour peak period. Concurrently, the robot-assisted group required fewer mannitol administrations, effectively preventing potential complications associated with prolonged use of dehydrating agents, such as electrolyte imbalances and renal impairment (25, 26).

Although the operative time was slightly shorter in the robot-assisted group, this difference did not reach statistical significance (*P* = 0.077). This may be attributed to the time required for robot registration and setup, which could offset the time saved from more precise puncture. As the team's experience with the robotic system grows, operative time may further decrease.

This study has several limitations that must be acknowledged. First, its retrospective, single-center, non-randomized design introduces inherent selection bias. Despite similar baseline characteristics, unmeasured confounders (e.g., exact time from ictus to surgery, subtle differences in preoperative care) may have influenced the results. Patients were assigned to groups based on robot availability and surgeon discretion, which could reflect a selection bias. Second, the relatively small sample size limits the statistical power and generalizability of our findings. No formal *a priori* sample size calculation was performed. Third, we did not perform multivariate regression analyses to adjust for potential confounding variables, which would strengthen the causal inference. Fourth, the follow-up period was limited to three months; longer-term functional and cognitive outcomes remain unknown. Fifth, as a single-center study, the results may not be directly transferable to institutions with different levels of expertise. Future multicenter, prospective, randomized controlled trials with larger sample sizes and longer follow-up are warranted to validate our findings.

In conclusion, Robot-assisted stereotactic minimally invasive puncture and aspiration for moderate basal ganglia hemorrhage provides more precise and thorough hematoma evacuation compared to conventional CT-guided puncture. This approach is associated with reduced perioperative morbidity, faster recovery, and improved short-term neurological and functional outcomes. While promising, these results require confirmation in larger, prospective, randomized studies.

## Data Availability

The original contributions presented in the study are included in the article/Supplementary Material, further inquiries can be directed to the corresponding author.
